# Neuroanatomy of the spleen: Mapping the relationship between sympathetic neurons and lymphocytes

**DOI:** 10.1371/journal.pone.0182416

**Published:** 2017-07-28

**Authors:** Kaitlin Murray, Dayn Romero Godinez, Ingrid Brust-Mascher, Elaine Nicole Miller, Melanie G. Gareau, Colin Reardon

**Affiliations:** Department. of Anatomy, Physiology, and Cell Biology, UC Davis School of Veterinary Medicine, UC Davis, Davis, California, United States of America; Weizmann Institute of Science, ISRAEL

## Abstract

The nervous system plays a profound regulatory role in maintaining appropriate immune responses by signaling to immune cells. These immune cells, including B- and T-cells, can further act as intermediary messengers, with subsets of B- and T-cells expressing choline acetyltransferase (ChAT), the enzyme required for acetylcholine (ACh) synthesis. Neural control of ACh release from ChAT^+^ T-cells can have powerful immune implications, regulating lymphocyte trafficking, inflammation, and prevent death due to experimental septic shock. Although ACh release from T-cells has been proposed to occur following norepinephrine (NE) released from sympathetic nerve terminals in the spleen, it is unknown how this communication occurs. While it was proposed that tyrosine hydroxylase (TH^+^) axons form synapse-like structures with ChAT^+^ T-cells, there is scant evidence to support or refute this phenomenon. With this in mind, we sought to determine the relative abundance of ChAT^+^ B- and T-cells in close proximity to TH^+^ axons, and determine what factors contribute to their localization in the spleen. Using confocal microscopy of tissue sections and three-dimensional imaging of intact spleen, we confirmed that ChAT^+^ B-cells exceed the number of ChAT^+^ T-cells, and overall few ChAT^+^ B- or T-cells are located close to TH^+^ fibers compared to total numbers. The organized location of ChAT^+^ lymphocytes within the spleen suggested that these cells were recruited by chemokine gradients. We identified ChAT^+^ B- and T-cells express the chemokine receptor CXCR5; indicating that these cells can respond to CXCL13 produced by stromal cells expressing the β2 adrenergic receptor in the spleen. Our findings suggest that sympathetic innervation contributes to organization of ChAT^+^ immune cells in the white pulp of the spleen by regulating CXCL13. Supporting this contention, chemical sympathectomy significantly reduced expression of this chemokine. Together, we demonstrated that there does not appear to be a basis for synaptic neuro-immune communication, and that sympathetic innervation can modulate immune function through altering stromal cell chemokine production.

## Introduction

Neural-immune interactions have long been observed to occur in numerous tissues that are critical for mediating immunological responses. Neurons are located in close proximity to B-cells, mast cells, macrophages, and T-cells in the intestinal mucosa and muscularis [1–3], skin [4], and secondary lymphoid organs including the spleen [5]. Recent studies have highlighted that these neural-immune interactions can dictate immunological outcomes, and that modulation of neuronal activity could become a new therapeutic modality in the treatment of immunopathologies [6].

Communication between the nervous system and immune cells has been previously demonstrated to reduce morbidity and mortality following overt immune responses in multiple diseases including arthritis, ulcerative colitis, ischemia-reperfusion injury, and septic shock via a the inflammatory reflex [7–12]. This reflex arc is initiated by detection of bacteria/bacterial products, or the resulting inflammatory processes by vagal afferent neurons. Activation of vagal afferents results in neuronal activation in the nucleus tractus solitarius, and after coordination in the brainstem, an efferent signal is conducted by the vagus nerve to the spleen. In the spleen, inhibition of aberrant immune responses requires ACh production by CD4^+^ T-cells that express choline acetyltransferase (ChAT) [5]. Stimulated release of ACh from CD4^+^ChAT^+^ T-cells occurs following activation of β2 adrenergic receptors (β2AR) by norepinephrine (NE). The source of this NE was proposed to be sympathetic neurons originating in the celiac ganglia and projecting into the spleen, although recent studies conducted in rat question the functional circuitry of this reflex arc [13]. Stimulated release of ACh from T-cells inhibits activation and TNFα production by red pulp, and marginal zone splenic macrophages during sterile endotoxemia [5].

The precise nature of the communication between sympathetic terminals and ChAT^+^ immune cells, as well as the frequency of ChAT^+^ T-cells intimately associated with these axons has not been resolved. ChAT expression in lymphocytes is not restricted to CD4^+^ T-cells; B-cells in both the spleen and lymph nodes comprise a significantly larger fraction of ChAT-GFP^+^ lymphocytes [14], in addition to small numbers of macrophages and dendritic cells [14, 15]. Despite this understanding of the cell types that can produce ACh, there is little information on the physical location of ChAT^+^ lymphocytes within secondary lymphoid organs, and the processes that determine this location.

Homing of specialized immune cells to discrete compartments within the spleen is a highly regulated process and is vital to the function of the immune system [16, 17]. This compartmentalization is afforded by production of chemotactic proteins or chemokines that serve to guide migration of cells expressing specific receptors. Although there are numerous chemokines present in the spleen, B-cells and T-cells predominantly respond to CXCL13 and CCL19/CCL21 gradients respectively [17–20]. Unique populations of stromal cells in the B- and T-cell zones producing these chemokines establish gradients to permit B- and T-cell homing. These include follicular dendritic cells (FDC) and marginal reticular cells that produce CXCL13 in B-cell zones [21, 22], and the fibroblastic reticular cells (FRC) producing CCL19/CCL21 in T-cell areas [19, 23]. Although these are the best characterized cell types, it is clear that additional stromal cells are present in secondary lymphoid organs, and that some of these cells express neurotransmitter receptors [24].

In support of a role for the nervous system in controlling stromal cell activity, sympathetic nerves play a vital role in the development of lymphoid tissue. Activation of nerve fibers that were identified as sympathetic, by virtue of tyrosine hydroxylase (TH^+^) expression, resulted in release of retinoic acid (RA), triggering CXCL13 production [25]. Despite these advances in neuro-immunology, the ability of the sympathetic innervation to regulate CXCL13 production in established secondary lymphoid organs is unknown.

With these questions in mind, we have performed confocal imaging studies over a large area of the spleen to determine the abundance of ChAT^+^ B and T-cells and their localization with respect to sympathetic nerves. This analysis has been supplemented using the CLARITY procedure [26–28] and multiphoton microscopy to provide detailed three-dimensional information on the relationship between TH^+^ axons and ChAT^+^ lymphocytes in the spleen. Using flow cytometry, the expression of CXCR5, the cognate receptor for CXCL13, was assessed on the surface of ChAT^+^ B- and T-cells, with identification of splenic stromal cells expressing CXCL13 and β2AR. The ability of sympathetic innervation to regulate CXCL13 expression was determined by chemical sympathectomy. In the spleen, sympathectomy significantly reduced CXCL13 expression, without affecting CCL19 or CCL21 expression. These findings indicate a previously unappreciated role of sympathetic innervation to alter immune responses in developed lymph nodes through maintaining chemokine production that recruit immune cells including ChAT^+^ B- and T-cells.

## Materials and methods

### Mice

ChAT-GFP (B6.Cg-Tg(RP23-268L19-EGFP)2Mik/J) and C57BL/6 mice were purchased from the Jackson laboratories (Bar Harbor, ME) and bred in-house. Male and female mice (6–8 weeks old) were euthanized by CO_2_ asphyxiation followed by cervical dislocation, according to AVMA guidelines. Chemical sympathectomy was performed by administration of 6-hydroxydopamine bromide (6-OHDA, Sigma-Aldrich, St Louis, MO.) by three i.p. injections (250 mg/kg or vehicle [PBS with 0.1% ascorbic acid]). Mice were euthanized 10 days after the last injection. All procedures were approved by the Institutional Animal Care and Use Committee at UC Davis.

### Confocal microscopy

Excised spleens were fixed in formalin for 24 h prior to graded dehydration in ethanol, embedded in paraffin, and cut on a microtome to produce 6 μm thick sections on slides. Slides were de-paraffinized and rehydrated according to standard protocols, and antigen retrieval was performed using citrate buffer (10 mM, pH 6.0, 1 h, 95°C). After blocking in 5% BSA normal donkey serum (1 h, room temperature), samples were incubated in primary antibody overnight (16 h 4°C). Primary antibodies used in this study were rabbit Anti-TH (Millipore, AB152, Billerica, MA), goat anti-GFP (Rockland Immunochemicals, Limerick, PA), and rat anti-CD3 (clone CD3-12, Bio-Rad, Hercules, CA), or rat anti-B220 (Abcam, Boston, MA.). After extensive washing (3 x 5mins), slides were incubated in appropriately labeled secondary antibodies (Invitrogen, Carlsbad, CA Table 1.) for 1 h at room temperature, washed and mounted in Prolong diamond (Invitrogen). Confocal imaging was performed on a Leica SP8 STED 3X microscope with a 63x 1.4 NA objective.

**Table 1:** Antibodies used for confocal and multiphoton microscopy.

**Antibody**	**Host**	**Source**	**Catalog No./clone**	**Dilution**	
Tyrosine Hydroxylase	Rabbit	Millipore	AB152	Confocal: 1:300 CLARITY: 1:100	
GFP	Goat	Rockland Immunochemicals	600-101-215	Confocal: 1:300 CLARITY: 1:100	
CD3	Rat	Bio-Rad	CD3-12	1:100	
B220	Rat	Abcam	RA3-6B2	1:100	
Goat anti-mouse CXCL13	goat	R&D Systems	AF470	1:50	
**Target**	**Host**	**Conjugate**	**Source**	**Catalog No.**	**Dilution**
Anti-goat	Donkey	Alexa Fluor488	Invitrogen	A11055	1:200
Anti-rabbit	Donkey	Alexa Fluor546	Invitrogen	A10040	1:200
Anti-rat	Donkey	Alexa Fluor647	Abcam	ab150155	1:200

### CLARITY imaging

Lymph nodes and spleens were immediately washed in PBS and immersed in CLARITY hydrogel monomer solution comprised of 4% formaldehyde (EMS Biosciences, Hatfield, PA), acrylamide, Bis-acrylamide (Bio-Rad) and the initiator VA-044 (Wako chemicals, Redmond VA). Tissues were immersed in this solution for 1 week at 4°C, followed by polymerization for 3 h at 37°C. Excess hydrogel was trimmed away and tissues were passively cleared for 3–4 weeks in clearing buffer (Boric Acid, SDS 4% v/v, pH 8.0, 37°C). Cleared samples were extensively washed in TBS-TritonX100 (0.1%v/v) for 1 week, and blocked for 48 h in TBS-TritonX100 (0.1% v/v) supplemented with 5% BSA (w/v, Sigma Aldrich St. Louis, MO) and normal donkey serum (Jackson Immunoresearch, West Grove, PA)

Tissue was subjected to immunostaining using rabbit anti-mouse TH (polyclonal, 1:100 [Millipore]) and goat anti-GFP (Rockland Immunochemicals). After incubation for 5 days, tissue samples were washed for 48 h at room temperature in TBS-TritonX100, and subjected to staining with anti-goat Alexa Fluor-488 and anti-rabbit Alex Fluor-546 conjugated secondary antibodies (Invitrogen). Following washing to remove unbound secondary antibody (24 h room temperature), samples were incubated in 85% glycerol for optical clearing (72 h room temperature).

Immunostained samples were imaged using a multiphoton microscope equipped with a spectral physics pulsed laser with group dispersion delay correction, reflected light hybrid detectors, and 25x 1.0 NA objective lens matched to the index of refraction of CLARITY samples (Leica Microsystems).

### Image analysis

Analysis of standard confocal data sets was performed by opening Leica image format files directly in FIJI [29]. Manual cell counts were aided by using the ‘cell counter’ pluggin. Analysis of cells within a set distance from TH^+^ fibers were performed by drawing line segments over the object of interest and expanding the selection to 8 μm from the object, and counted manually with the ‘cell counter’. Determining the abundance of CXCL13 expression in the spleen was performed by creation of surfaces in Imaris (v8.2 Bitplane scientific, Concord, MA) around the CXCL13 immunoreactive cells. The volume of these objects was then determined and divided by the volume of the splenic white pulp that was imaged.

CLARITY raw data was converted to the Imaris image format using the Imaris file converter (Bitplane scientific, Concord, MA), and filtered using a median Gaussian filter (3 x 3x 1 pixels) in Imaris 8.2. Tiled images were aligned using XUVTools [30] and montages imported into Imaris 8.2. Models of nerve fibers were created using the “surface” tool after subdividing the volume into X volumes while lymphocytes were modeled in a similar manner using the “spot” tool. Distances to the nearest TH^+^ nerve fiber were calculated by using MatLab (Mathworks, Natick, MA), and are available as supplementary information **(S1 File)**.

### Stromal cell isolation

Stromal cells were isolated using modifications to previously published protocols [31, 32]. Briefly, excised spleens were macerated using scissors placed in RPMI media containing 0.8 mg/ml Dispase, 0.2 mg/ml collagenase P and 0.1 mg/ml DNase I and incubated in a shaker incubator (20 mins, 37°C, 300 rpm). Released cells and remaining tissue fragments were passed through a 100 μm cell strainer, with released cells collected in the flow through and added to PBS containing 2% FBS and 5 mM EDTA on ice. Tissue fragments were digested further by incubation in fresh enzyme containing media. This process was repeated three times, and dissociated cells from each digestion were pooled for analysis by flow cytometry.

### Flow cytometry

Lymphocytes were isolated by passage through a 100 μm cell strainer (BD Biosciences, San Jose, CA). Single cell suspensions were subjected to red blood cell lysis by resuspension in ACK buffer. Following blocking of cell surface Fc receptors with anti-CD16/32, lymphocytes were surface stained using anti -CD4, -B220, -PD-1, -CXCR5. Cell viability was determined using Live/dead violet. Stromal cells were identified by staining with anti -CD45-PE, -CD31-PE-Cy7 in stain buffer (20 min, 4°C, **Table 2**). After extensive washing to remove unbound antibodies, cells were subjected to fixation and permeabilization using Fix/Perm reagent according to manufacturer’s instructions (BD Biosciences). Intracellular staining was performed using rabbit anti-β2AR (Abcam) [33–35] followed by donkey anti-rabbit BV421 (Biolegend, San Diego, CA) and goat anti-CXCL13 (RnD Systems, Minneapolis, MN) followed by donkey anti-goat Alex Fluor 647 (Invitrogen). After extensive washing to remove unbound antibodies, flow cytometry was performed on a LSRII running DIVA 6.0 (BD Biosciences). Data was analyzed using FlowJo v10.0 (Treestar, Ashland, Oregon).

**Table 2:** Antibodies used for flow cytometry.

Antibody	Source	Conjugate	cat#/clone	Dilution
anti-mouse CD16/32 "Fc block"	Tonbo Biosciences	N/A	2.4G2	1:200
anti-mouse CD4	BD Biosciences	PerCP-Cy5.5	RM4-5	1:200
anti-mouse B220	BD Biosciences	APC	RA3-6B2	1:200
anti-mouse PD-1	BD Biosciences	BV605	J43	1:200
anti mouse CXCR5	BD Biosciences	PE	2G8	1:200
anti mouse CD45	BD Biosciences	PE	30F11	1:200
anti-mouse CD31	BD Biosciences	PE-Cy7	390	1:200
rabbit anti-mouse b2AR	Abcam	N/A	ab182136/ EPR707(N)	1:100
goat anti-mouse CXCL13	R&D Systems	N/A	AF470	1:100
donkey anti-rabbit	Biolegend	BV421	406410	1:200
donkey anti-goat	Invitrogen	AlexaFluor647	A21447	1:200

### Quantitative PCR

Analysis of gene expression was performed by real time quantitative PCR as previously described [14]. Briefly, excised spleens were homogenized in Trizol (Invitrogen) using a 5 mm stainless steel bead (Qiagen) in a bead beater. RNA was extracted as directed by manufacturer’s instructions, with isolated RNA dissolved in ultrapure H_2_0 (Invitrogen). Synthesis of cDNA was performed using an iSCRIPT reverse transcriptase kit (Bio-Rad, Hercules, CA), and real time qPCR was performed for the following targets using the indicated primer pairs from Primerbank [36]: *Th* forward: 5’- GTGCCAGAGAGGACAAGGTTC-3’, reverse: 5’- CGATACGCCTGGTCAGAGA-3’, *Cxcl13* forward: 5’- GGCCACGGTATTCTGGAAGC-3’, reverse: 5’- GGGCGTAACTTGAATCCGATCTA-3’, *Ccl19* forward: 5’- GGGGTGCTAATGATGCGGAA-3’, reverse: 5’- CCTTAGTGTGGTGAACACAACA-3’, *Ccl21* forward: 5’- GTGATGGAGGGGGTCAGGA-3’, reverse 5’- GGGATGGGACAGCCTAAACT-3’, *Actb* forward: 5’-GGCTGTATTCCCCTCCATCG-3’, reverse: 5’- CCAGTTGGTAACAATGCCATGT-3’. Amplification and data acquisition was conducted using a QuantStudio6 (Thermo Fisher scientific, Waltham, MA). Data were analyzed using the delta deltaCT method normalizing gene expression to *Actb* in each sample followed by normalization to experimental control sample.

### Statistical analysis

Data were analyzed using a two-tailed t-test in Prism (Graphpad, San Diego CA), with a P value of less than 0.05 denoted as significant.

## Results

### Most ChAT^+^ immune cells are not closely juxtaposed to TH^+^ axons in the spleen

**Fig 1:**
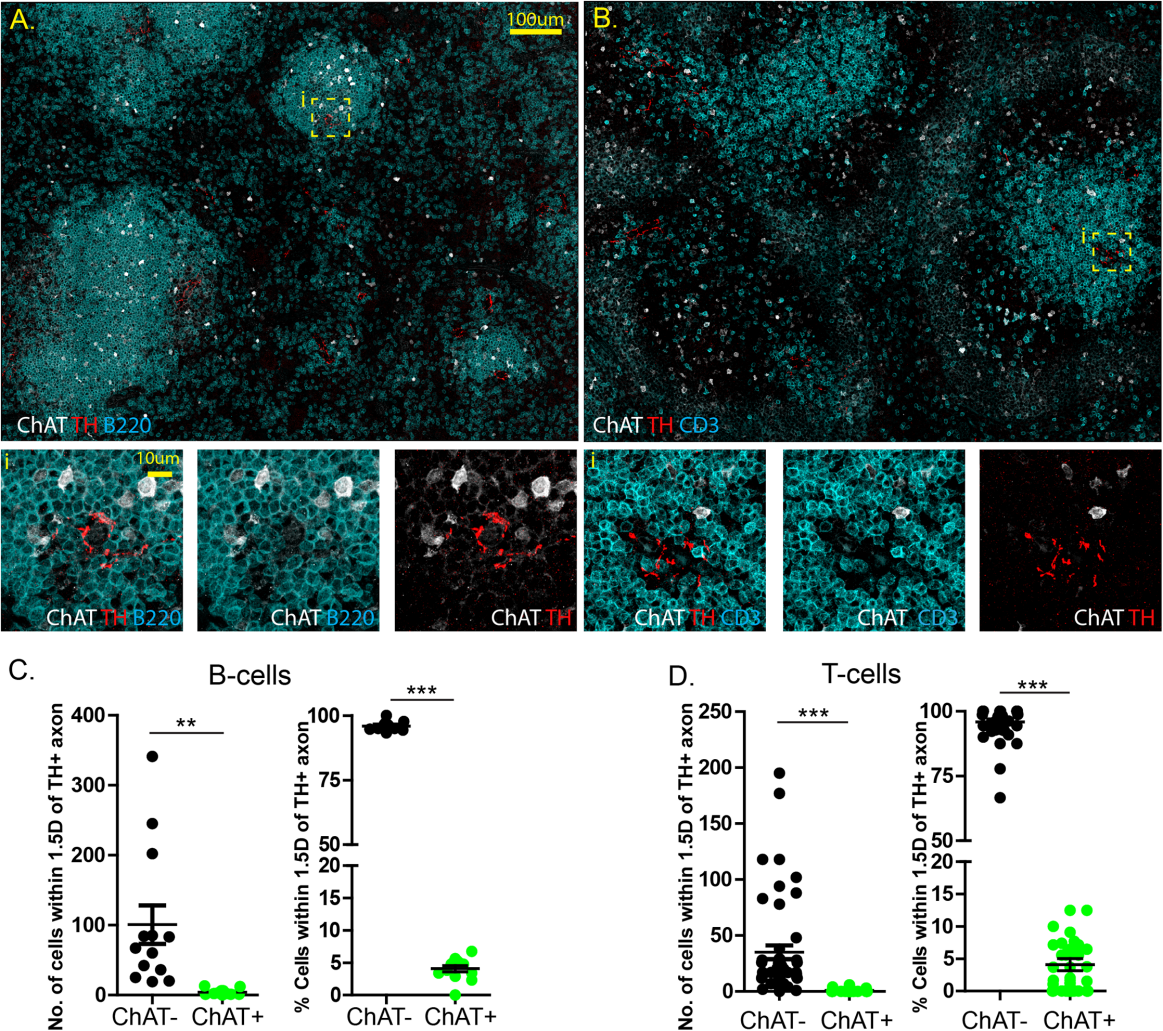
Interactions between splenic ChAT^+^ lymphocytes and TH^+^ axons are rare events. Confocal microscopy was conducted on spleens from ChAT-GFP reporter mice with immunostaining for CD3, ChAT, TH **(A),** or B220, ChAT, TH **(B)**. Quantification of the total number of ChAT^+^ and ChAT^-^ B-cells **(C)** and T-cells **(D)** with in 1.5 cell diameters. ** P<0.001, *** P<0.0001.

To assess the number of ChAT^+^ B and T-cells that are closely juxtaposed to sympathetic terminals in the spleen, immunostaining was performed for TH, GFP, and B220 or CD3 respectively **(Fig 1A & 1B)**. Using this approach, exceedingly few ChAT^+^ B220^+^ B-cells or ChAT^+^ CD3^+^ T-cells were observed near TH^+^ axons. While the majority of ChAT^+^ lymphocytes were identified as B220^+^ B-cells and not CD3^+^ T-cells, these cells were not closely localized to TH^+^ axons. Quantification reveled that ChAT-GFP^+^ B220^+^ B-cells represent 4.08 ± 0.47% of B-cells (B220^+^) within 1.5 fold the average lymphocyte diameter, or 8 μm, of a TH^+^ axon **(Fig 1A & 1C)**. Similarly, CD3^+^ ChAT-GFP^+^ T-cells were only 4.09 ± 0.92% of all T-cells within 8 μm of a TH^+^ axon **(Fig 1B & 1D)**. Together, these data indicate that ChAT^+^ B- and T-cells are rare events and not likely to be intimately associated with TH^+^ axons compared to ChAT^-^ B- or T-cells.

**Fig 2:**
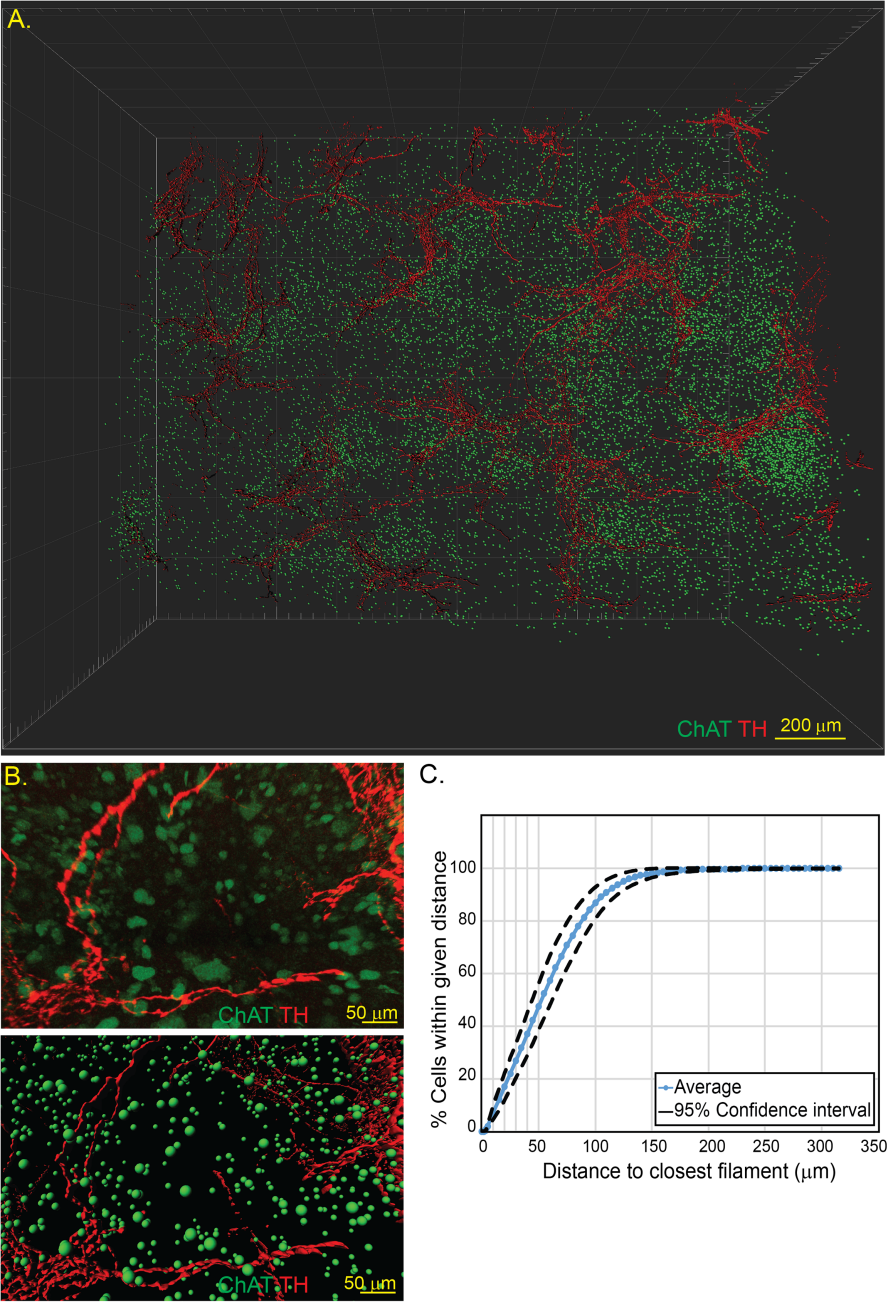
Three-dimensional reconstruction of splenic neuro-immune interactions by CLARITY. Spleens from ChAT-GFP mice were subjected to CLARITY and imaged by two-photon microscopy **(A)** allowing for individual cells and neural surfaces to be identified and modelled **(B)**. Distances of ChAT^+^ lymphocytes to the nearest TH^+^ axons was determined and quantified **(C)**. Representative images from n = 6 animals.

We considered that given the relatively large volume of the spleen relative to the small sampling in histological sections, lack of ChAT^+^ lymphocyte–neural interactions could be due to under-sampling. Consequently, we employed the CLARITY procedure permitting imaging of the intact spleen, with three-dimensional reconstruction of the imaged organ. Spleens from ChAT-GFP^+^ mice were rendered optically transparent using the CLARITY process and immunostained for GFP and TH, to reveal the position of ChAT^+^ cells and TH^+^ axons **(Fig 2A)**. Computerized reconstruction of the imaged volume was performed to identify and model ChAT^+^ lymphocytes and TH^+^ axons allowing quantitative data to be extracted **(Fig 2B & 2C)**. Using this approach, the distance from the center of each ChAT^+^ cell to the surface of the nearest TH^+^ axon was calculated. In spleens from six naïve ChAT-GFP mice, the frequency of cells within 5 μm (i.e. one cell diameter) of a TH^+^ neuronal fiber was found to be 1% **(Fig 2C)**. This methodology performed on multiple ChAT-GFP mice raise the possibility that although there is some biological inter-subject variation, the distribution of ChAT^+^ lymphocytes in the spleen may not be a random process.

### CXCR5 is expressed by ChAT^+^ B- and T-cells

**Fig 3:**
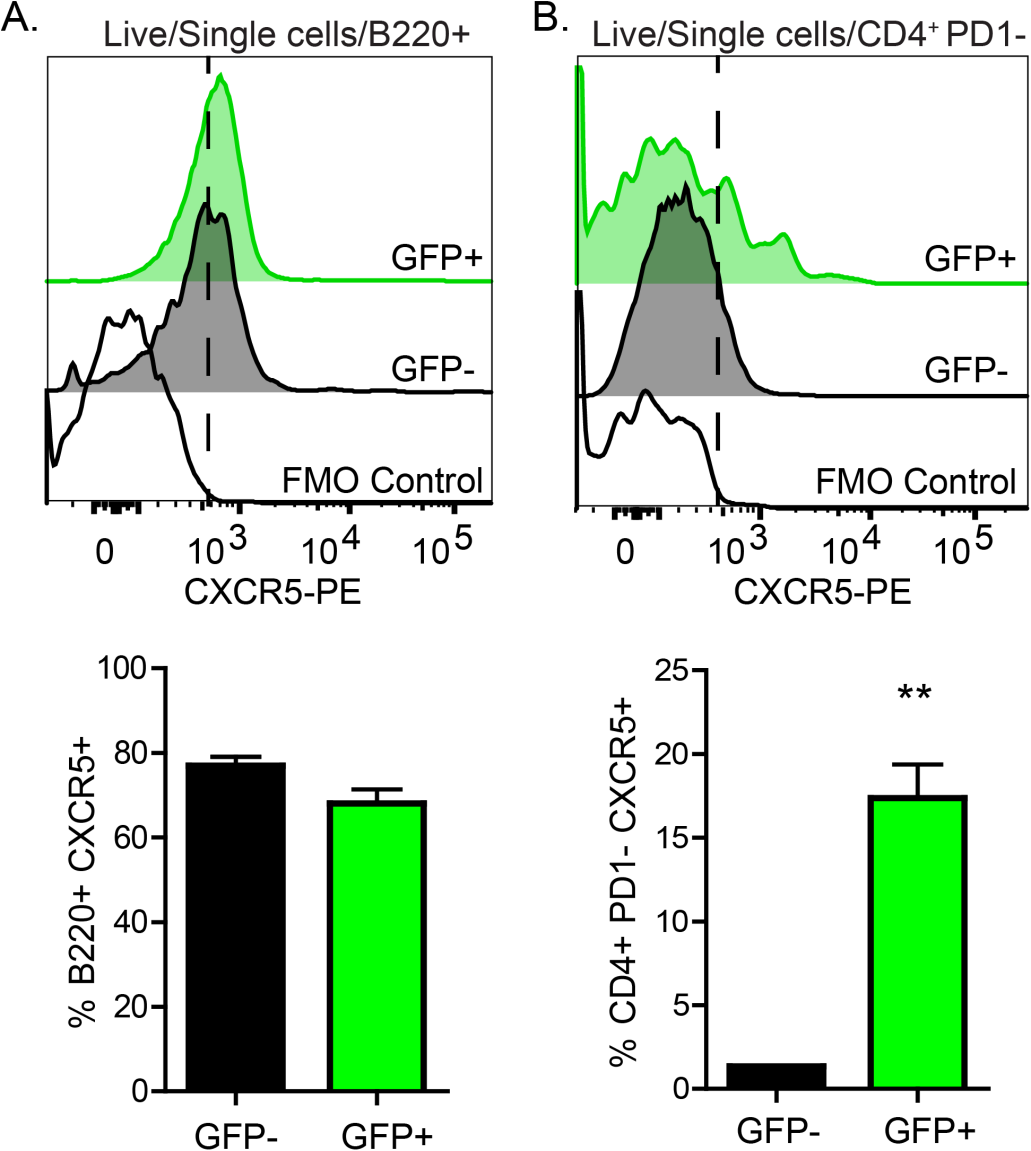
ChAT^+^ B- and T-cells express CXCR5. Splenocytes isolated from ChAT-GFP reporter mice were subjected to flow cytometry to assess CXCR5 expression on ChAT^+^ and ChAT- B^-^ **(A)**, and T-cells **(B)**. **P<0.01, n = 6 mice.

Considering the higher frequency of ChAT^+^ B- and T-cells in the B-cell zones of the splenic white pulp, we considered that these cells could respond to CXCL13 by expression of CXCR5. Flow cytometric analysis conducted on splenocytes from naïve ChAT-GFP mice demonstrated that ChAT^+^ B-cells and T-cells express CXCR5 **(Fig 3A & 3B)**. While expression of CXCR5 in ChAT^-^ and ChAT^+^ B-cells was not different, ChAT^+^ T-cells expressed significantly more CXCR5 compared to ChAT^-^ T-cells **(Fig 3A & 3B, lower panels)**. It is important to note that ChAT-GFP^+^ T-cells expressing CXCR5 were PD-1^-^, indicating that they were unlikely to belong to the T follicular helper cell lineage.

### CXCL13 producing splenic stromal cells express β2AR

**Fig 4:**
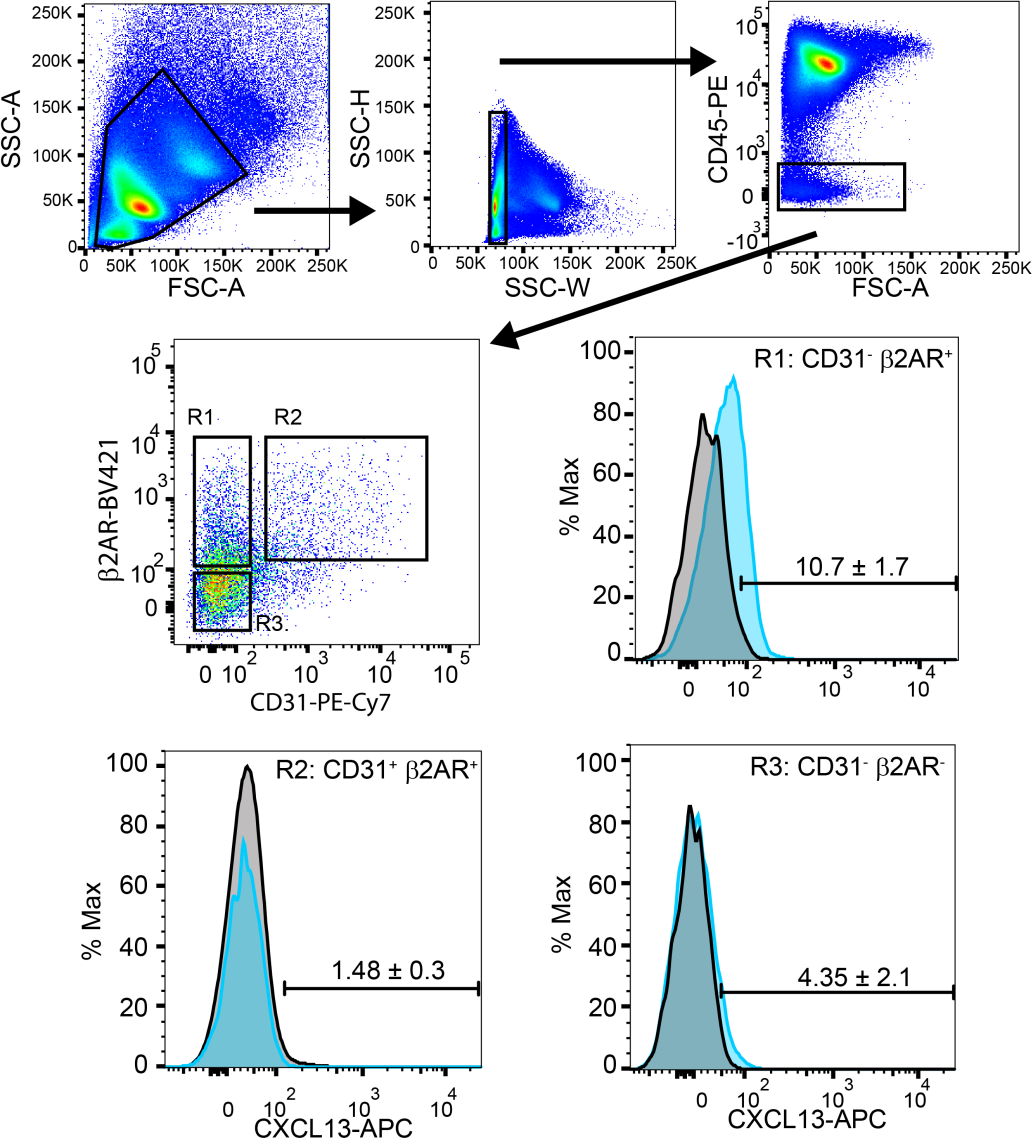
Splenic stromal cells express β2AR and CXCL13. The expression of β2AR and CXCL13 in stromal cells isolated from C57BL/6 mice were characterized by flow cytometry. Stromal cells were identified using the gating strategy depicted to include single cells, CD45^-^ (top panels). Stromal cells were then identified based on lack of CD31, with β2AR^+^ (R1) and β2AR^-^ (R3) populations assessed for CXCL13. Data are representative from 4 mice, mean ± SD.

To assess how signals from the sympathetic nervous system could be conveyed to splenic stromal cells, flow cytometry was used to assess the expression of β2AR and CXCL13^+^ **(Fig 4)**. Splenic stromal cells were identified using a gating strategy to identify single cells that were negative for CD45 expression **(Fig 4)**. These cells were further characterized based on the expression of CD31, a marker of endothelial cells, and β2AR. These three cell populations; the non-endothelial CD31^-^ β2AR^+^ and CD31^-^ β2AR^-^ cells, and the endothelial CD31^+^ β2AR^+^ cells were assessed for CXCL13 expression. CXCL13 was predominantly detected in the CD31 β2AR^+^ cells compared to CD31^-^ β2AR^-^ cells **(Fig 4 “R1” vs. “R2”)**. As expected, although β2AR was expressed on endothelial cells, CXCL13 was not detected. These data demonstrate that splenic stromal cells express β2AR, produce CXCL13 and could respond to sympathetic neurotransmitters such as NE.

### Expression of CXCL13 is controlled by the splenic sympathetic innervation

**Fig 5:**
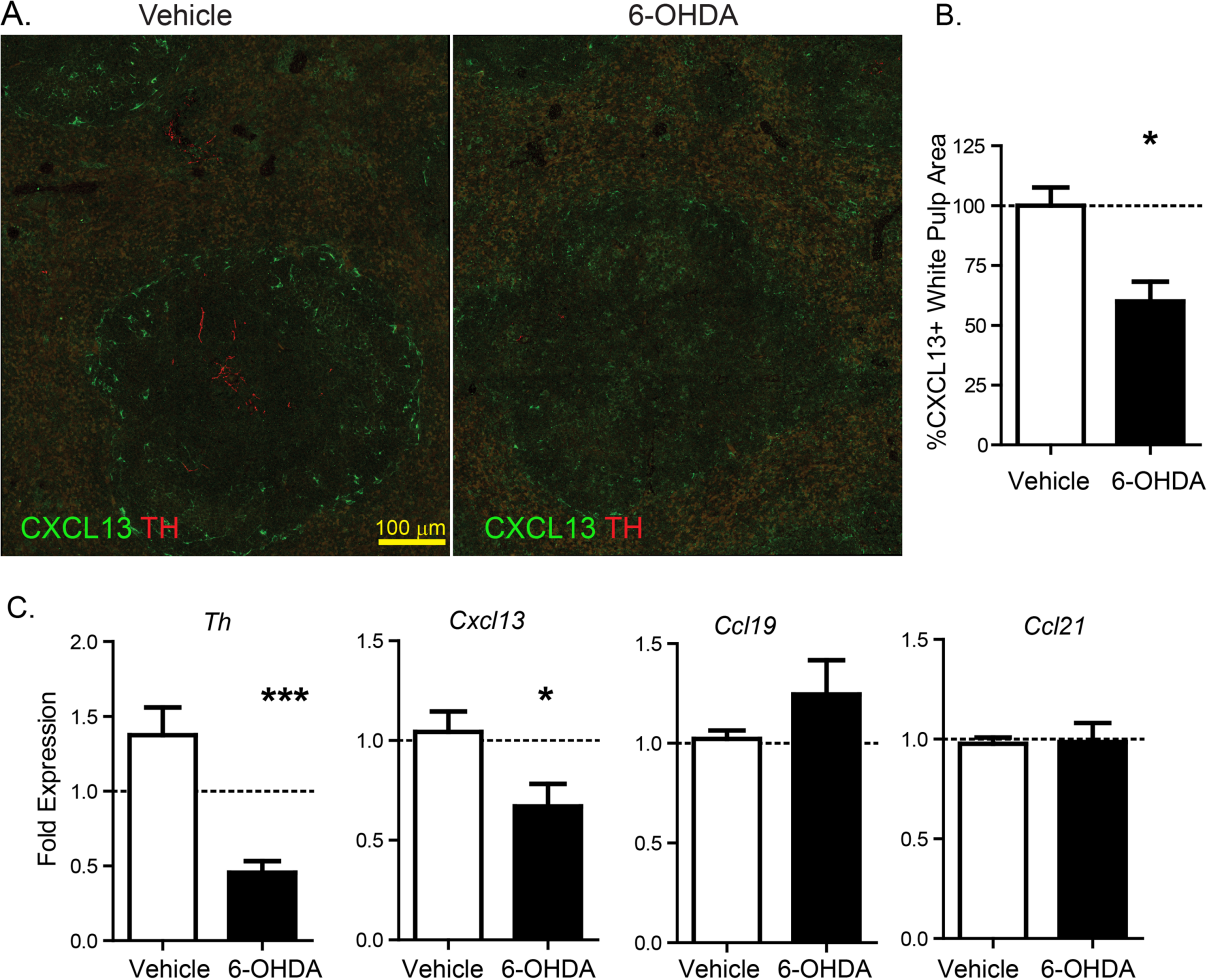
Splenic sympathetic innervation regulates CXCL13 expression. Spleen from control mice or mice subjected to chemical sympathectomy by 6-OHDA administration were assessed for expression of TH and CXCL13 by confocal microscopy **(A)** and quantified **(B)**. Expression of *Th*, *Ccl19*, C*cl21* and *Cxcl13* expression by qRT-PCR. *P<0.05, *** P<0.001.

Confocal imaging studies indicating TH^+^ immunoreactive axons in B-cell zones, coupled with our identification of CXCL13^+^ stromal cells that express β2AR, led us to determine the influence of sympathetic innervation on splenic chemokine production in naïve mice. To determine if sympathetic innervation was capable of regulating CXCL13 expression, mice were subjected to chemical sympathectomy using 6-OHDA treatment. Ten days after chemical sympathectomy, there was a significant reduction in TH^+^ splenic axons and CXCL13 expression as determined by quantification of confocal microscope images **(Fig 5A & 5B)**.

Regulation of *Cxcl13* expression in spleen from vehicle or 6-OHDA treated mice were assessed by qRT-PCR. Chemical sympathectomy significantly reduced the expression of *Th* and the chemokine *Cxcl13* compared to vehicle control, with no significant change in the expression of the T-cell chemokines *Ccl19* and *Ccl21* **(Fig 5C)**. These data suggest that sympathetic innervation help maintain expression of specific chemokines that are critical to the organization and location of ChAT^+^ B and T-cells within the spleen.

## Discussion

Neural modulation of immune function has emerged as an unexpected mechanism that can prevent severe immunopathology. Despite the ability for neuronal signaling to inhibit macrophage activation, and prevent death in models of septic shock, the biological basis for this communication remains unclear [13, 37]. Although postganglionic sympathetic neurons in the periphery terminate in target organs and often lack synaptic contact with target cells [38], a physical interaction has been suggested to occur between the TH^+^ axons and ChAT^+^ T-cells in the spleen [5]. This was based on the observations of close physical approximation, or “synaptic-like” interactions between these axons and lymphocytes by transmission electron microscopy [39]. As these observations were not conducted using a stereological approach and are limited to a small area of the spleen, it is not possible to determine the abundance of these interactions. Our confocal analysis performed over a large region of the spleen highlights the rarity of ChAT-GFP^+^ CD3^+^ T-cells relative to the more abundant ChAT-GFP^+^ B220^+^ B-cell population. Furthermore, confocal analysis suggested that this rare population did not appear more likely than CD3^+^ChAT-GFP^-^ T-cells to be closely juxtaposed to TH^+^ sympathetic innervation. By using CLARITY and multiphoton imaging to reconstruct a large volume of the spleen in a non-biased approach, we have observed that there are few ChAT^+^ lymphocytes within 1.5 cell diameters (8 μm), let alone within the 15–20 nm that would constitute a traditional synapse [40, 41]. Perhaps equally contentious is the apparent neuro-immune synapses that were reported, with lymphocytes directly adjacent to TH^+^ nerve fibers but also endothelial cells. Indeed, our data suggest that ChAT^+^ lymphocytes are no more likely to be adjacent to a TH^+^ axons in the spleen compared to ChAT^-^ cells, suggesting that there is no inherently unique direct physical connection with ChAT^+^ cells. Critically, the lack of synaptic-like interactions should not be equated to a lack of neuro-immune communication in the spleen, as classical synapses are not prerequisite for neural signaling [42–46]. Catecholaminergic neurotransmitters such as NE have been observed in neuronal signaling through diffusion or “volume transmission” in the central nervous system [43, 45]. In the periphery, sympathetic axons terminate within the target tissue without specialized post-junctional structures in place, and require diffusion of neurotransmitters before reaching target cell bearing a receptor. These non-synaptic structures have a functional consequence with a slower rate of neurotransmission compared to synapse [38]. We propose that this mechanism could also function as the mechanism of communication between ChAT^+^ T-cells and sympathetic innervation in the spleen. Further experiments used to determine ChAT+ T-cell activation following neural stimulation will be fundamental to increasing our knowledge of this system.

The functional ramifications of a synapse-like or direct physical interaction versus a diffusion or “volume transmission” model are vast. A direct interaction model could imply that CD4+ChAT+ T-cells home to axons, form intimate associations, and if these were maintained would cease Immunosurveillance of dendritic cells. This tight association would suggest that unless released from synapse-like structures, CD4^+^ChAT^+^ T-cells would have limited mobility, would fail to circulate, thereby exerting a highly localized regulatory effect. In a diffusion-based model, the relationship between axons, CD4^+^ChAT^+^ cells, and effector immune cells will also be of vital importance in modeling non-synaptic communications between the constituents of the neuro-immune network. In such a model, diffusion of neurotransmitters, and therefore the distance between cells, will dictate the quantity of signal received. Lack of static positioning of ChAT^+^ T-cells within the spleen could also imply that after activation by signals from the nervous system, these cells could migrate to and effect distance tissues and physiological states including blood pressure [47].

Our quantitative three-dimensional modelling of the neuro-immune architecture of the spleen further revealed surprisingly little inter-subject variation. Both ChAT^+^ B- and T-cells in the spleen expressed CXCR5, suggesting that these cells are responsive to CXCL13 chemokine gradients produced by specialized splenic stromal cells. These data suggest that homing to specific chemokines could control the distribution of ChAT^+^ B- and T-cells in the spleen. Despite expression of this chemokine receptor and finding of ChAT^+^ T-cells in B-cell zones, the functional implications of these cells are unclear. While electrical stimulation of the vagus nerve can reduce B-cell production of antibodies directed against *Streptococcus pneumoniae* antigens, this is a classical T-independent B-cell response [48]. Moreover, CD138^+^ antibody secreting cells were found to localize with splenic nerve fibers in the red pulp [48], a region that is devoid of ChAT^+^ T-cells in naïve mice. As such, it is uncertain if ChAT^+^ T-cells residing in close proximity to B-cells in naïve mice aid in antibody production, or if there are several neuro-immune pathways that regulate different aspects of B-cell function depending on the type of antigen response.

Organization of lymphoid organs and homing of immune cells to the appropriate physical regions are vital for immunological function. The sympathetic innervation of the spleen contributes to this organization by maintaining CXCL13 expression by a sub-population of stromal cells. Significantly reduced CXCL13 expression was observed following chemical sympathectomy. This ability of sympathetic innervation to induce CXCL13 expression has been previously established during development; where neuronal activation and release of RA-induced CXCL13 expression from stromal cells occurs in developing lymph nodes [25]. While it is tempting to speculate that RA from sympathetic nerve terminals acts on stromal cells to induce homing of ChAT^+^ T-cells, this could be one of many signals acting in concert to regulate lymphocyte homing. For example, CXCL13 expression is synergistically increased when follicular dendritic cells within lymph nodes and intestinal Peyer’s patches are stimulated with RA and TLR agonists compared to RA or TLR agonists alone [49]. In accordance with a previous report [24], we have confirmed that CXLC13 producing stromal cells express the β2AR, a feature that could permit these cells to respond to NE released by the sympathetic innervation in adult mice. These data are not in opposition to the canonical pathway of lymphotoxin-induced CXCL13 expression, but rather suggest that additional signals from a variety of sources, including sympathetic innervation, can aid immune development and homeostasis.

Therefore, we have identified that ChAT^+^ B and T cells are rare events in the spleen, and the majority of these cells are not close enough to TH^+^ axons to form synapse-like interactions. Further studies delineating signaling molecules from neurons that alter immune organ structure and functionality may well serve as new therapeutic targets to modulate the formation of tertiary lymphoid tissues and relieve immunopathology.

## Supporting information

S1 File Distance measurements from the ChAT-GFP^+^ lymphocytes to the surface of a TH^+^ axon. Using Imaris, ChAT-GFP^+^ lymphocytes and the surfaces of axons using the built-in “spots” and surfaces modeling tools. Distance from the modeled ChAT-GFP^+^ lymphocytes and the closest surface of a TH^+^ neuron were calculated using MatLAB. Histogram bins were constructed and the frequency of cells within that distance were calculated. Results were averaged for six mice.
(XLSX)
